# The complete chloroplast genome and phylogenetic analysis of *Fraxinus paxiana* Lingelsh. 1907 (Oleaceae)

**DOI:** 10.1080/23802359.2025.2573760

**Published:** 2026-01-05

**Authors:** Hong Feng, Wei Sun, Xiangxiao Meng, Shilin Chen

**Affiliations:** ^a^ School of Chinese Materia Medica, Tianjin University of Traditional Chinese Medicine, Tianjin, China; ^b^ Institute of Chinese Materia Medica, China Academy of Chinese Medical Sciences, State Key Laboratory for Quality Ensurance and Sustainable Use of Dao-di Herb, Beijing, China; ^c^ Institute of Chinese Materia Medica, China Academy of Chinese Medical Sciences, Key Laboratory of Beijing for Identification and Safety Evaluation of Chinese Medicine, Beijing, China; ^d^ Institute of Herbgenomics, Chengdu University of Traditional Chinese Medicine, Chengdu, China

**Keywords:** *Fraxinus paxiana*, complete chloroplast genome, phylogenetic analysis

## Abstract

*Fraxinus paxiana* distributed in Shaanxi, Gansu, Hubei, Hunan, and Sichuan provinces*,* is used as a local medicine in Shaanxi and also serves as an adulterant of traditional Chinese medicine Qinpi. Here, we assembled its 155,692 bp chloroplast genome (GC content 37.87%) with a typical quadripartite structure. A total of 133 genes were annotated, including 88 protein-coding genes, 37 tRNA genes, and eight rRNA genes. We identified 43 SSRs, predominantly composed of mononucleotide repeats. Phylogenetic analysis showed that the genus *Fraxinus* is divided into two branches, with *F. paxiana* and *F. insularis* having the closest phylogenetic relationship.

## 1. Introduction

The genus *Fraxinus* Linnaeus belongs to the Oleaceae family and comprises about 60 species worldwide and 27 species in China of deciduous trees or rarely shrubs (Guo et al. 2025). Some species are widely used in commercial timber and landscaping, among which *Fraxinus mandschurica* is a well-known species for commercial timber production (Xie et al. 2023). In addition, some species in this genus have important medicinal value. In the Chinese Pharmacopoeia, the traditional Chinese medicine (TCM) Qinpi is the dried branch bark or stem bark of *F. rhynchophylla*, *F. chinensis*, *F. szaboana*, or *F. stylosa*, mainly used to clear heat and dry dampness, astringe the intestines to stop dysentery, vaginal discharge, and improve vision (Sarfraz et al. 2017; Guo et al. 2025).

*Fraxinus paxiana* Lingelsh. 1907 (Oleaceae) is distributed in the valleys, slopes, and sparse forests of Shaanxi, Gansu, Hubei, Hunan, and Sichuan provinces. The stem bark of *F. paxiana* is often used locally to clear heat, dry dampness, converge and stop bleeding, but it is not an officially recognized origin in the Pharmacopoeia (Ma and Zhao 2008; Zhao et al. 2025). As a result, it is often sold as a counterfeit of Qinpi, which poses potential risks to the efficacy and safety of TCM. Currently, research on *F. paxiana* remains limited, with few studies focusing on its chemical composition and phylogenetic relationships. To date, all phylogenetic studies of *F. paxiana* have relied on combinations of molecular markers (Wallander 2012; Hinsinger et al. 2013; Dupin et al. 2024). The chloroplast genome is an important resource for species identification and phylogenetic analysis (Guo et al. 2023; Wang et al. 2025), but the chloroplast genome of *F. paxiana* has not yet been reported. This study successfully assembled and annotated the complete chloroplast genome of *F. paxiana*, and conducted a phylogenetic analysis of the genus *Fraxinus*. This study provides a valuable genomic resource for the taxonomy and conservation of *Fraxinus* species, and offers a molecular basis for identifying counterfeit Qinpi.

## 2. Materials and methods

**Figure 1.**
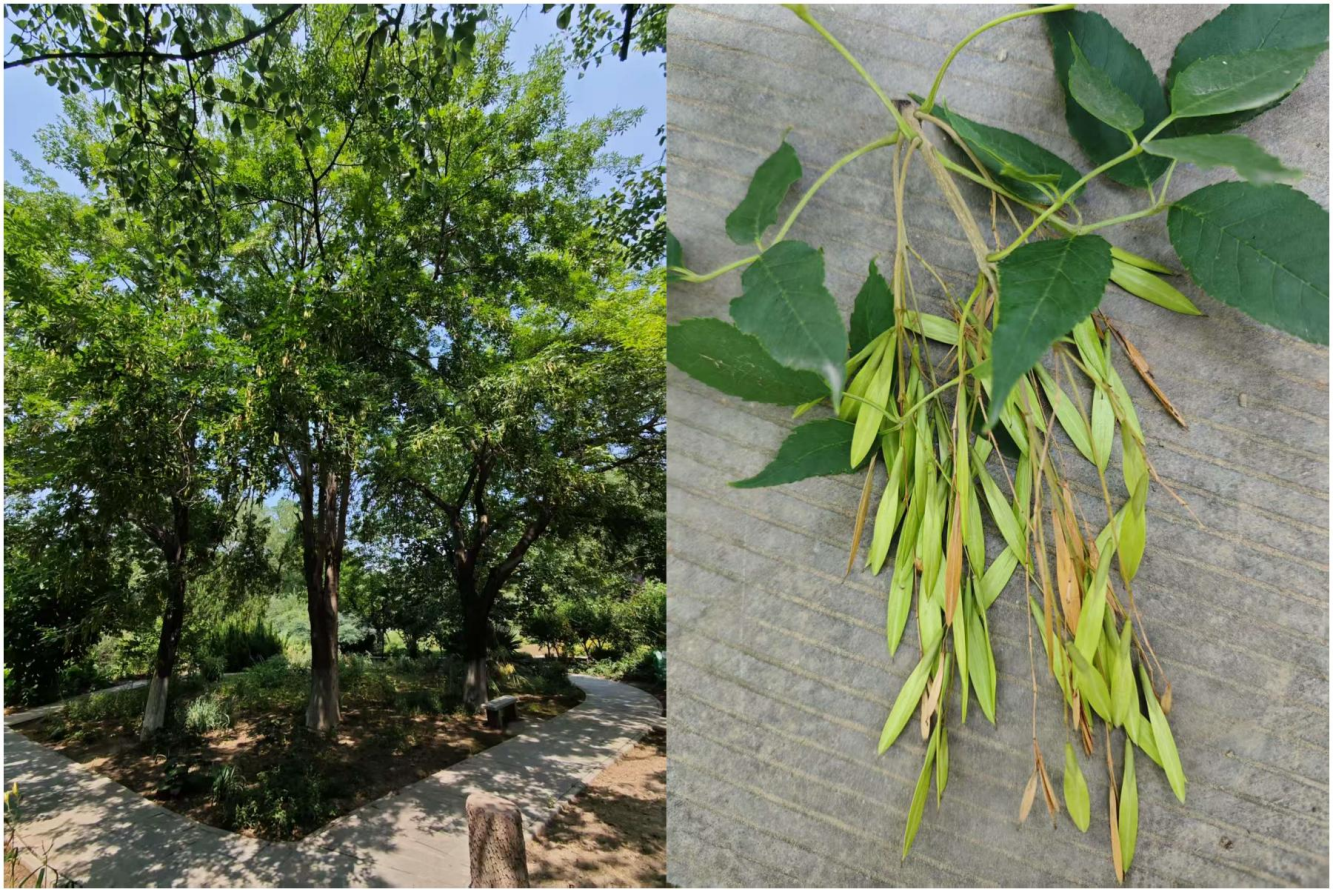
The morphological characteristics of *F. paxiana. Left*: Trees up to 20 m with trunk diameter at breast height of 1 m. *Right*: Leaflets 7–9; petiolule 0–2 mm; leaflet blade lanceolate to ovate-oblong, length 5–18 cm, width 2–6 cm, papery, base rounded to attenuate, margin crenate, apex acuminate; primary veins 2–16 on each side of midrib. Samara linear-spatulate, length 2.5–3 cm; width 4 mm; wing decurrent to upper part of nutlet. The photos of the species were taken by Hong Feng.

Fresh leaves of *F. paxiana* were collected from Xian County, Shaanxi Province, China (Figure 1; 34.208611°N, 108.953056°E) and dried them using silica gel for DNA extraction. The voucher specimen was identified by Hong Feng and was deposited at Tianjin International Joint Academy of Biomedicine (voucher number FPX20200625-3; Hong Feng, fenghong@tjab.org). Genomic DNA was extracted from these dry leaves using the NuClean Plant Genomic DNA Kit (CWBIO, Taizhou, China). DNA quantity was determined using Qubit^®^ 3.0 Fluorometer (Life Technologies, Carlsbad, CA). DNA sequencing was carried out using the Illumina NovaSeq 6000 with 150 bp paired-end (Illumina, San Diego, CA). The raw data were filtered using fastp version 0.20.0 (Chen et al. 2018) with default parameters, yielding 4.46 GB of clean reads. The complete chloroplast genome of *F. paxiana* was assembled using GetOrganelle v1.7.7.1 (Jin et al. 2020) with default parameters. The complete chloroplast genome was annotated using CPGAVAS2 (Shi et al. 2019), and manually checked using Geneious Prime (Biomatters Ltd., Auckland, New Zealand). The chloroplast genome map was visualized using OGDRAW (Greiner et al. 2019). The clean reads were mapped to the chloroplast genome using BWA v0.7.17 (Li 2013), and Samtools v1.9 (Li et al. 2009) was employed to detect the coverage depth. The schematic representations of cis-splicing and trans-splicing genes were generated using CPGView (Liu et al. 2023). Simple sequence repeats (SSRs) of chloroplast genome were identified using the SSRs sub-commands of Cpstools version 2.6 (Huang et al. 2024).

For phylogenetic analysis, the complete chloroplast genomes of 17 species of the genus *Fraxinus* were downloaded from the GenBank, with *Olea europaea* (NC_013707) designated as the outgroup. The common protein-coding genes (PCGs) of the 19 chloroplast genomes were extracted using phy sub-commands of Cpstools version 2.6 (Huang et al. 2024), and aligned using MAFFT v7.450 (Katoh and Standley 2013) with default parameters. The maximum-likelihood (ML) tree of the 19 species was reconstructed in IQ-TREE version 2.1.2 (Minh et al. 2020) with 1000 ultrafast bootstrap replicates. The phylogenetic tree was then visualized by Figtree v1.4.4 (http://tree.bio.ed.ac.uk/software/figtree/).

## 3. Results

**Figure 2.**
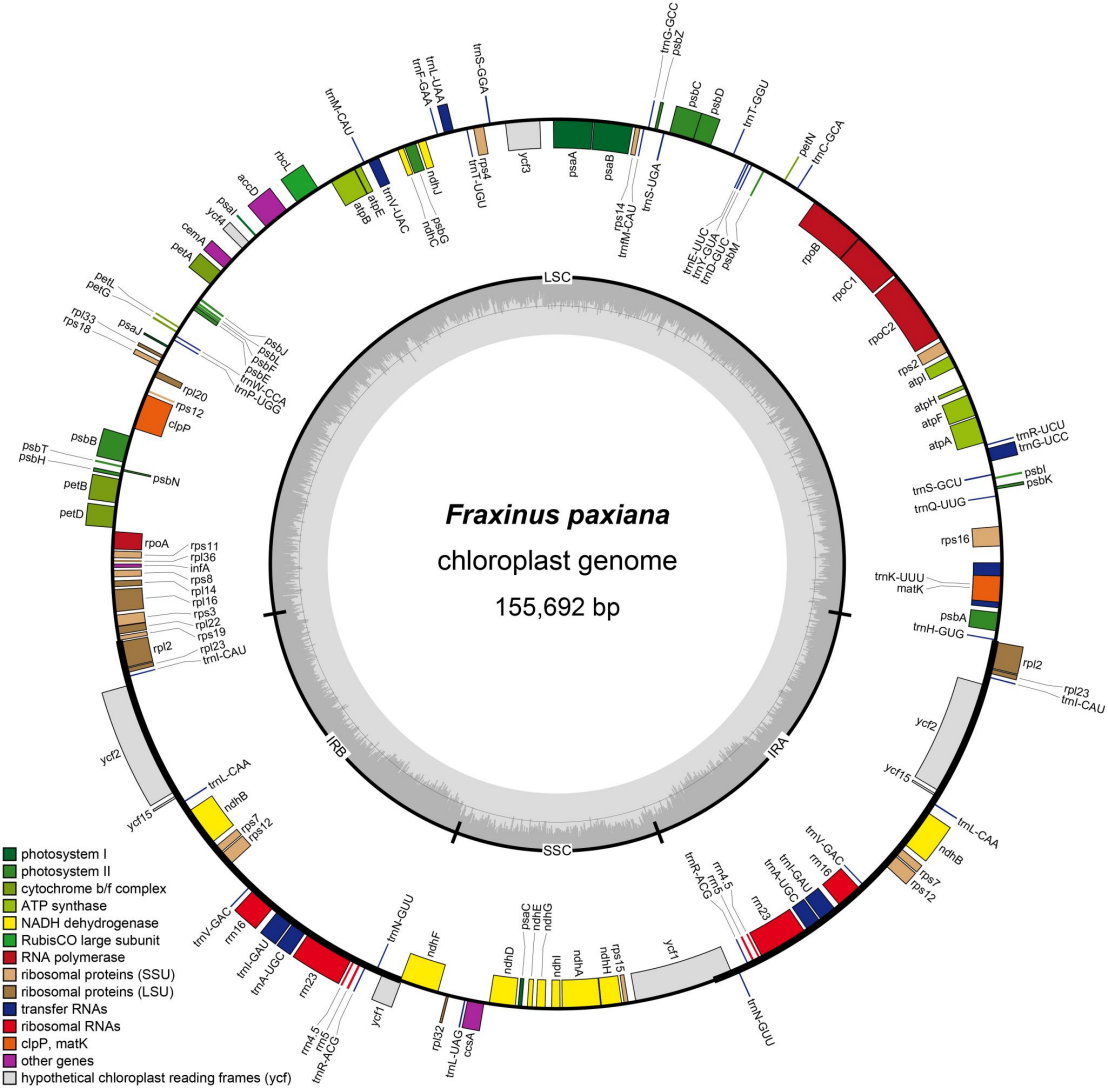
The circular map of *F. paxiana* chloroplast genome, generated by OGDraw. In the innermost circle, darker grey corresponds to GC content while the lighter grey corresponds to AT content. In the outer circle, genes on the outside are transcribed clockwise, while genes on the inside are transcribed counterclockwise. Genes belonging to different functional groups are color-coded. IR: inverted repeat; LSC: large single-copy region; SSC: small single-copy region.

The complete chloroplast genome of *F. paxiana* exhibits a typical quadripartite structure, with a total length of 155,692 bp and a GC content of 37.87% (Figure 2). The complete chloroplast genome comprises a large single-copy region (LSC, 86,442 bp), a small single-copy region (SSC, 17,844 bp), and two inverted repeat regions (IR, 25,703 bp) (Figure 2). The minimum and average coverage depth of the genome assembly were 511× and 2693.04× (Figure S1). The annotated genome contained 133 genes in total, including 88 PCGs, 37 tRNA genes, and eight rRNA genes. Nineteen genes were duplicated in the IR regions, including eight PCGs, seven tRNA genes, and four rRNA genes. Among these, nine PCGs (*atp*F, *ndh*A, *ndh*B, *pet*B, *pet*D, *rpl*16, *rpl*2, *rpoC*1, *rps*16) contain an intron, while two PCGs (*clp*P, *ycf*3) contain two introns and were cis-splicing genes (Figure S2). Moreover, *rps*12 gene was identified as a trans-splicing gene with three exons (Figure S3). In this study, a total of 43 SSRs were detected in *F. paxiana* chloroplast genome, with mononucleotide repeats being the most abundant (41 out of 43 SSRs).

**Figure 3.**
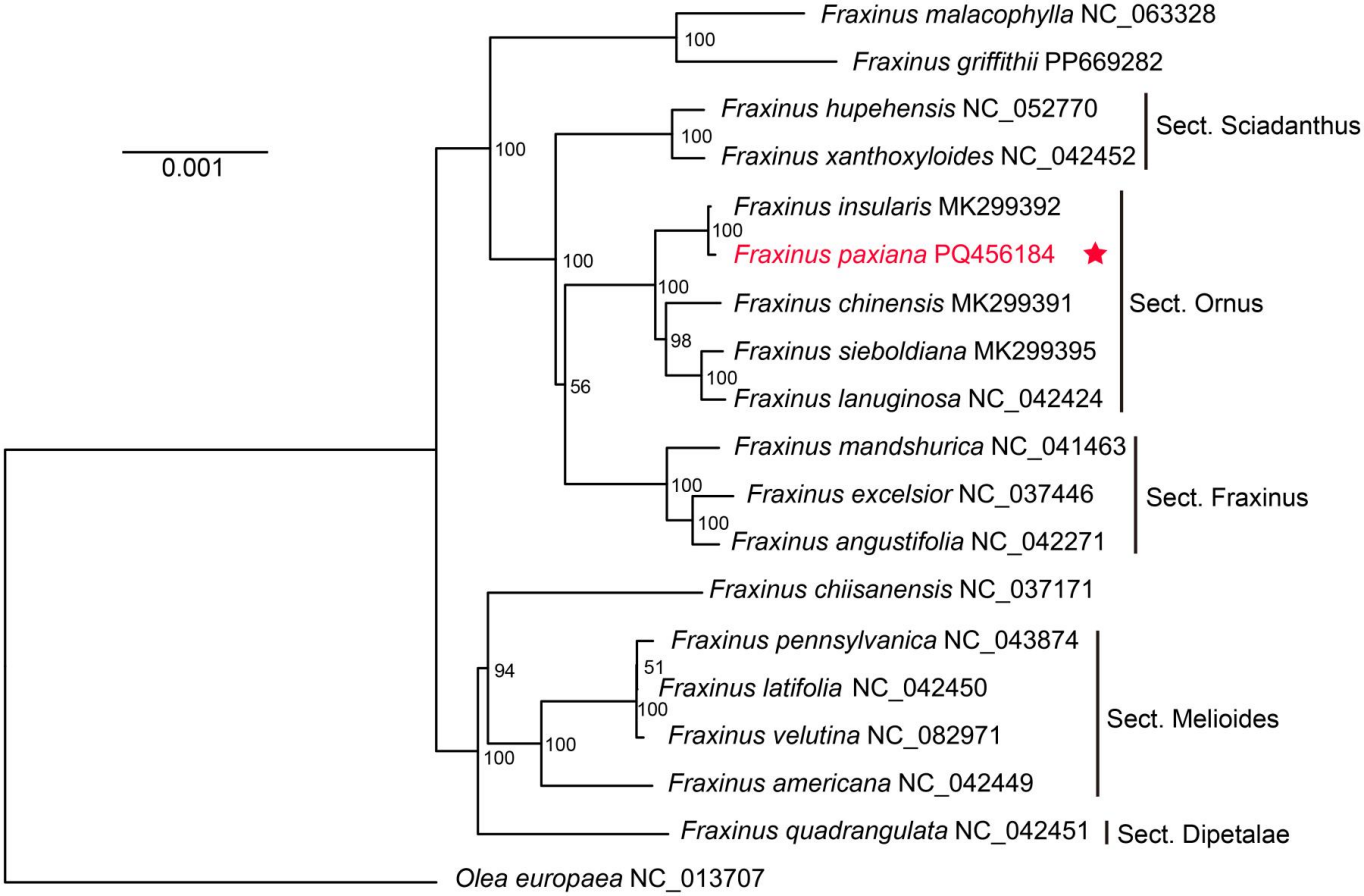
Maximum-likelihood tree based on the chloroplast gene sequences of *F. paxiana* and 18 other species using the maximum-likelihood method. The number next to the nodes indicates the bootstrap values. The chloroplast genomes of *F. paxiana* in this study were labeled in red and marked with a red star. The sequences are used in phylogenetic analysis consist of *F. xanthoxyloides* NC_042452, *F. insularis* MK299392, *F. chinensis* MK299391, *F. sieboldiana* MK299395, *F. lanuginosa* NC_042424, *F. americana* NC_042449, *F. quadrangulata* NC_042451, *F. angustifolia* NC_042271, *F. latifolia* NC_042450 (Olofsson et al. 2019), *F. malacophylla* NC_063328 (Duan et al. 2020), *F. hupehensis* NC_052770 (Zhang et al. 2020), *F. mandshurica* NC_041463 (Zhao et al. 2018), *F. chiisanensis* NC_037171 (Kim et al. 2019), *F. pennsylvanica* NC_043874 (Yi et al. 2019), *F. excelsior* NC_037446 (unpublished), *F. velutina* NC_082971 (unpublished), *F. griffithii* PP669282 (unpublished), and *O. europaea* NC_013707 (unpublished).

The coding sequences of 70 shared PCGs from 19 chloroplast genomes were analyzed to determine the phylogenetic placement of *F. paxiana*. In the phylogenetic tree, 18 *Fraxinus* species can be classified into two main branches, largely consistent with previous studies (Olofsson et al. 2019). Most branches in the phylogenetic tree showed strong support, with bootstrap values exceeding 80%. Within this framework, *F. paxiana* was resolved as a sister species to *F. insularis*, which is consistent with previous reports (Dupin et al. 2024).

## 4. Discussion and conclusions

In this study, the complete chloroplast genome of *F. paxiana* was reported for the first time, containing 133 genes, including 88 PCGs, 37 tRNA genes, and eight rRNA genes. Among them, 19 pairs of genes were located in IRs. The chloroplast genome of *F. paxiana* is similar in size and structure to those of other reported *Fraxinus* species, which indicates a relatively conserved chloroplast genome in this genus. Moreover, a total of 43 SSRs were detected in the chloroplast genome of *F. paxiana*. The polymorphism of SSRs can provide a basis for molecular identification of the genus *Fraxinus*. The phylogenetic analysis showed that genus *Fraxinus* is divided into two main branches, which is similar to the findings of Olofsson et al. (2019). Among them, *F. paxiana* has the closest genetic relationship with *F. insularis.* This study provides new insights into the phylogenetics and resource conservation of the genus *Fraxinus*, and establishes a molecular basis for the identification of Qinpi and its adulterants.

## Supplementary Material

Supplemental Material

Supplemental Material

Supplemental Material

## Data Availability

The genome sequence data that support the findings of this study are openly available in GenBank of NCBI at https://www.ncbi.nlm.nih.gov under the accession number PQ456184. The associated BioProject, SRA, and Bio-Sample numbers are PRJNA1289343, SRR34467720, and SAMN49887613, respectively.
