# Supplementary figures and images for: The complete chloroplast genome and phylogenetic analysis of Fraxinus paxiana Lingelsh. 1907 (Oleaceae)
Source: Mitochondrial DNA B Resour. 2026 Jan 5;11(2):232–6. doi: 10.1080/23802359.2025.2573760 (PMC12777748; doi:10.1080/23802359.2025.2573760)

# Trans-splicing Genes

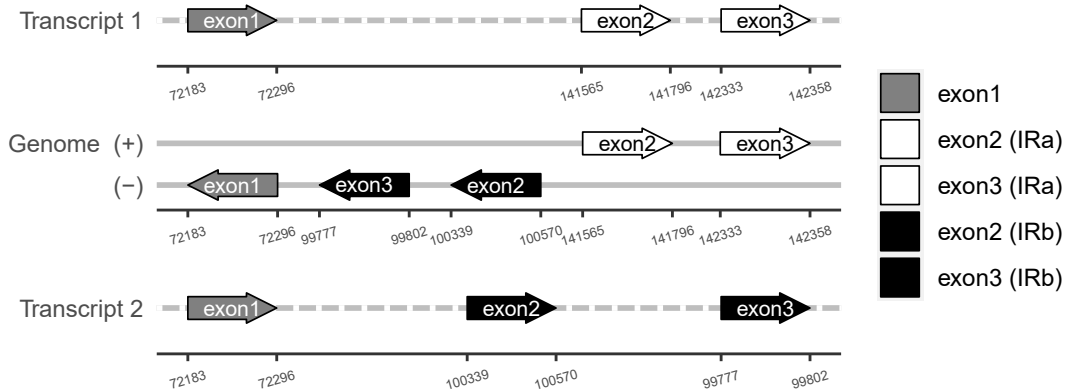

Supplement: Supplemental Material [file TMDN_A_2573760_SM0258.pdf]

# Cis-splicing Genes

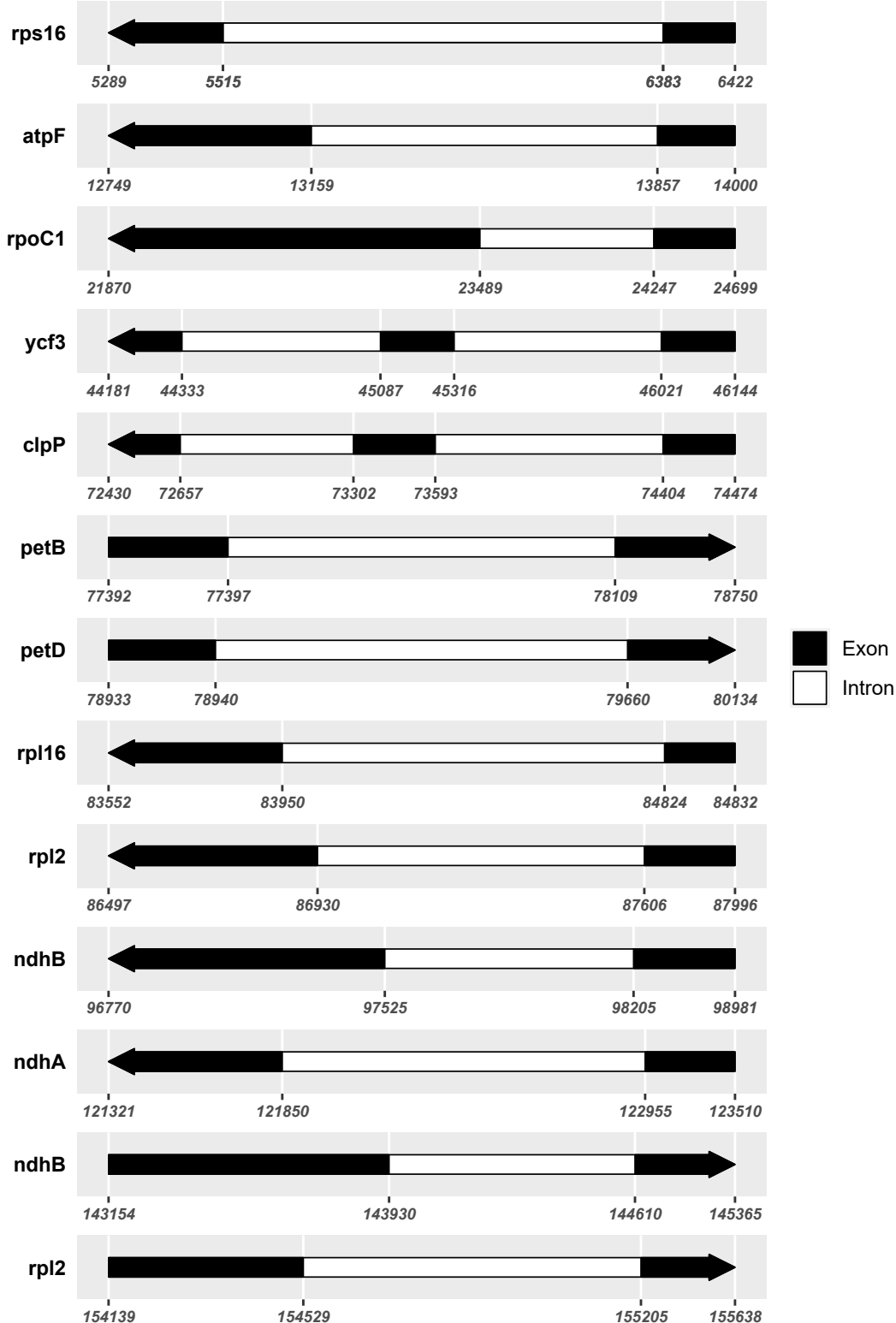

Supplement: Supplemental Material [file TMDN_A_2573760_SM0253.pdf]

Sequencing Depth and Coverage Map

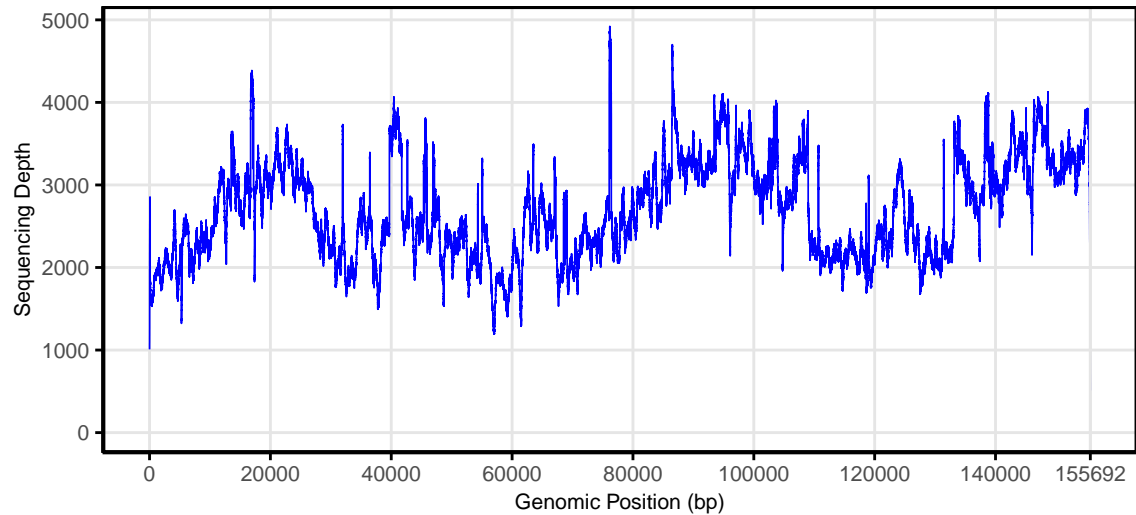

Supplement: Supplemental Material [file TMDN_A_2573760_SM0249.pdf]
